# Peri‐Implant Radiographic Measurements Compared With Intra‐Surgical Assessments: Diagnostic Accuracy and Reproducibility

**DOI:** 10.1111/jre.70169

**Published:** 2026-09-29

**Authors:** Era Kakar, Parham Hazrati, Andrea Laureti, Zhaozhao Chen, Hom‐Lay Wang

**Affiliations:** ^1^ Department of Periodontics and Oral Medicine, School of Dentistry University of Michigan Ann Arbor Michigan USA; ^2^ Periodontics and Implantology Advanced Computing and Artificial Intelligence (PICO.AI) Ann Arbor Michigan USA; ^3^ Department of Oral Medicine, Infection, and Immunity Harvard School of Dental Medicine Boston Massachusetts USA; ^4^ Division of Fixed Prosthodontics and Biomaterials, University Clinics for Dental Medicine University of Geneva Geneva Switzerland; ^5^ Department of Chemical Science and Technologies University of Rome Tor Vergata Rome Italy

**Keywords:** calibration, defect angle, defect height, defect width, emergence angle, inter‐examiner reliability, intra‐examiner reliability, periapical radiographs, peri‐implantitis, radiographic measurement

## Abstract

Not all peri‐implant radiographic measurements. Across eight calibrated examiners, emergence angle and defect width were highly reproducible, but defect height, defect angle, and distal bone levels varied widely and underestimated the true defect size measured intrasurgically. Calibration and multi‐examiner averaging matter.

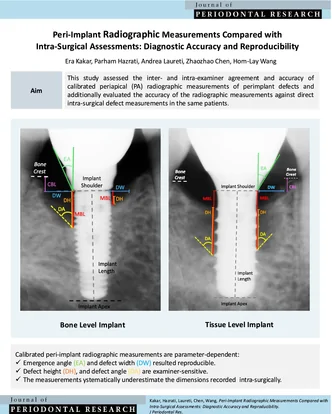

## 1 Introduction

Peri‐implant radiographic measurements increasingly inform clinical and research decision‐making, yet their reproducibility has been established mainly for marginal and crestal bone levels (MBL and CBL) [1, 2]. Newer parameters: restoration emergence angle (EA) and emergence profile (EP), peri‐implant defect width (DW), height (DH), and angle (DA) are now routinely reported and used in peri‐implantitis decision‐making [3, 4], yet their inter‐ and intra‐examiner reliability and their agreement with true surgical dimensions remain largely undefined. We assessed the inter‐ and intra‐examiner agreement of calibrated periapical (PA) radiographic measurements for all the above reported parameters and additionally evaluated the accuracy of the radiographic measurements of DW and DH against direct intra‐surgical defect measurements in the same patients.

## 2 Methods

This cross‐sectional study (University of Michigan Institutional Review Board HUM00255314) was reported in accordance with the STROBE guidelines. Ten de‐identified PA radiographs were obtained with the cone paralleling technique with the Extension Cone Paralleling System (Dentsply Rinn, Elgin, IL, USA), a size‐2 intraoral sensor (GXS‐700, KaVo Dental/Gendex, Biberach, Germany), and standardized exposure parameters (70 kV/7 mA/0.25 s) from patients with peri‐implantitis defects (bone‐ and tissue‐level implants) evaluated at least 12 months after functional loading and completion of initial bone remodeling, drawn from a registered clinical study (HUM00169735) in which intra‐surgical defect dimensions of DW and DH had been recorded. All patients had provided written informed consent for use of de‐identified data.

The workflow comprised four sequential phases. In the training phase, eight graduate periodontics fellows were instructed by an expert (H‐L.W.) who defined and demonstrated each parameter—MBL, CBL, EA, EP, DW, DH, and DA (Figure 1, Table S2), in ImageJ (National Institutes of Health, Bethesda, MD, USA); five separate radiographs, not part of the study set, were used for training. Every image was then calibrated to the known implant length to correct for distortion. In three subsequent measurement sessions held at 2‐week intervals, all examiners independently measured the same study radiographs while blinded to their own and to one another's previous values; each parameter was measured three times and averaged. The complete workflow is described in Table S1. Two investigators (E.K., P.H.) compiled the data. Intra‐surgical measurements of DW and DH were obtained with a UNC periodontal probe (Hu‐Friedy Mfg. Co., Chicago, USA) following full‐thickness flap elevation at the time of surgery. DH was measured from the bone crest to the implant platform (bone‐level implants) or to the transition to the endosseous portion of the fixture (tissue‐level implants); DW was measured in the plane perpendicular to the implant long axis, between the most coronal point of the endosseous part of the implant and the bone crest (Figure S1).

**FIGURE 1:**
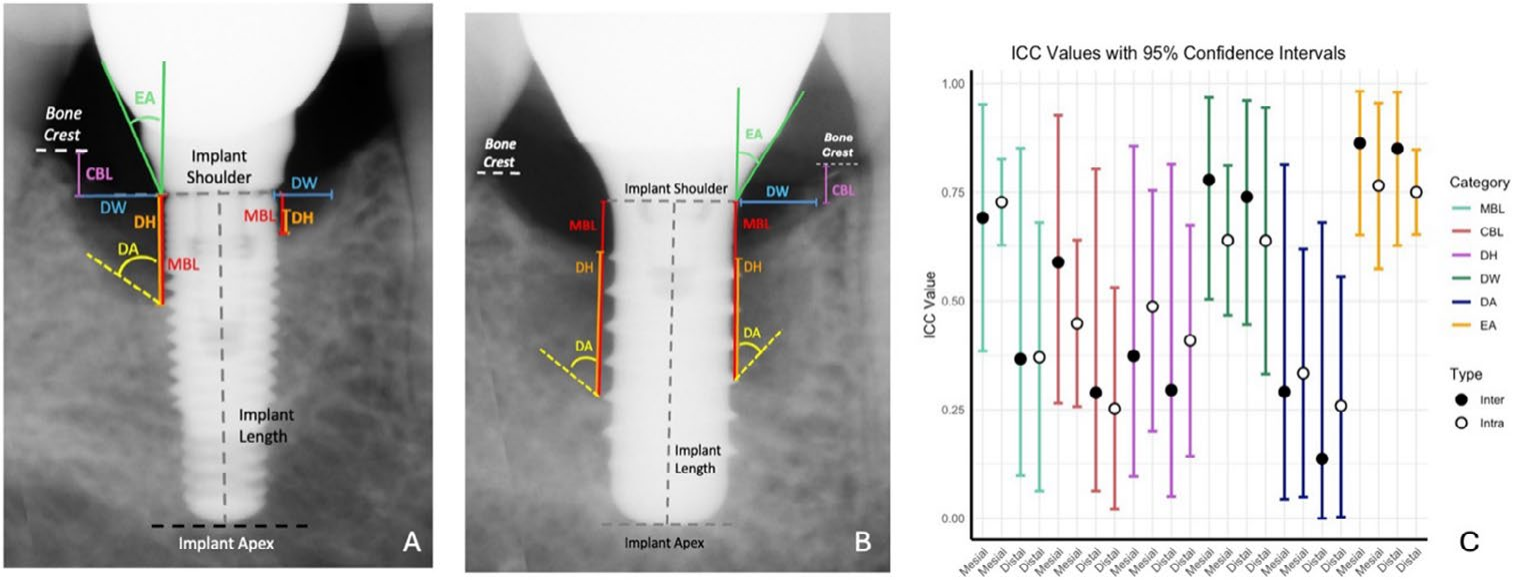
Calibrated peri‐implant radiographic measurement parameters and their examiner reliability. (A, B) Two representative periapical radiographs of a Bone Level (A) and a Tissue Level (B) implant illustrating the parameters measured at the mesial and distal aspects of the implant, referenced to the bone crest, implant shoulder, implant length, and implant apex: marginal bone level (MBL), crestal bone level (CBL), defect height (DH), defect width (DW), defect angle (DA), and restoration emergence angle (EA). (C) Inter‐examiner (filled circles) and intra‐examiner (open circles) intraclass correlation coefficient (ICC) values, with 95% confidence intervals, for each continuous parameter at the mesial and distal sites.

Two‐way mixed‐effects intraclass correlation coefficients (ICC), reported with their 95% confidence intervals to reflect the precision afforded by the sample size, assessed continuous variables; Fleiss' *κ* assessed categorical variables (EP; bone‐ vs. tissue‐level connection); and radiographic‐surgical agreement of the DH and DW parameters was evaluated with the Pearson correlation coefficient, mean absolute error, and Bland–Altman analysis (Rstudio; *α* = 0.05). Agreement was graded as poor (< 0.40), fair (0.40–0.59), good (0.60–0.74), or excellent (0.75–1.00). The sample size was determined by the availability of cases with complete intra‐surgical documentation in the parent registered study rather than by an a priori power calculation; this study was therefore designed as an exploratory, hypothesis‐generating pilot analysis. Full statistical modeling details, image calibration procedure, and operational definitions of all radiographic parameters are provided in the Data S1.

## 3 Results

Inter‐examiner reliability was excellent for EA and mesial DW and good for distal DW (ICC = 0.739), while mesial CBL was only fair; distal MBL, distal CBL, mesial and distal DH, and mesial and distal DA showed poor agreement, with distal DA not reaching significance. Intra‐examiner reliability was excellent for EA and good for mesial MBL and DW, whereas DA remained poor. For the categorical comparison of bone‐ versus tissue‐level implants, agreement was good (inter *κ* = 0.625; intra *κ* = 0.719), but EP agreement was poor (*κ* < 0.40).

Compared with intra‐surgical measurements, radiographs tended to underestimate defect dimensions: mean differences were −2.99 mm (mesial) and −3.52 mm (distal) for DH and −0.883 mm (mesial) and −0.463 mm (distal) for DW; the mesial DH difference was not statistically significant. Surgical and radiographic DH were significantly but only moderately correlated (mesial *r* = 0.479; distal *r* = 0.342; both *p* < 0.001) (Figure 2).

**FIGURE 2:**
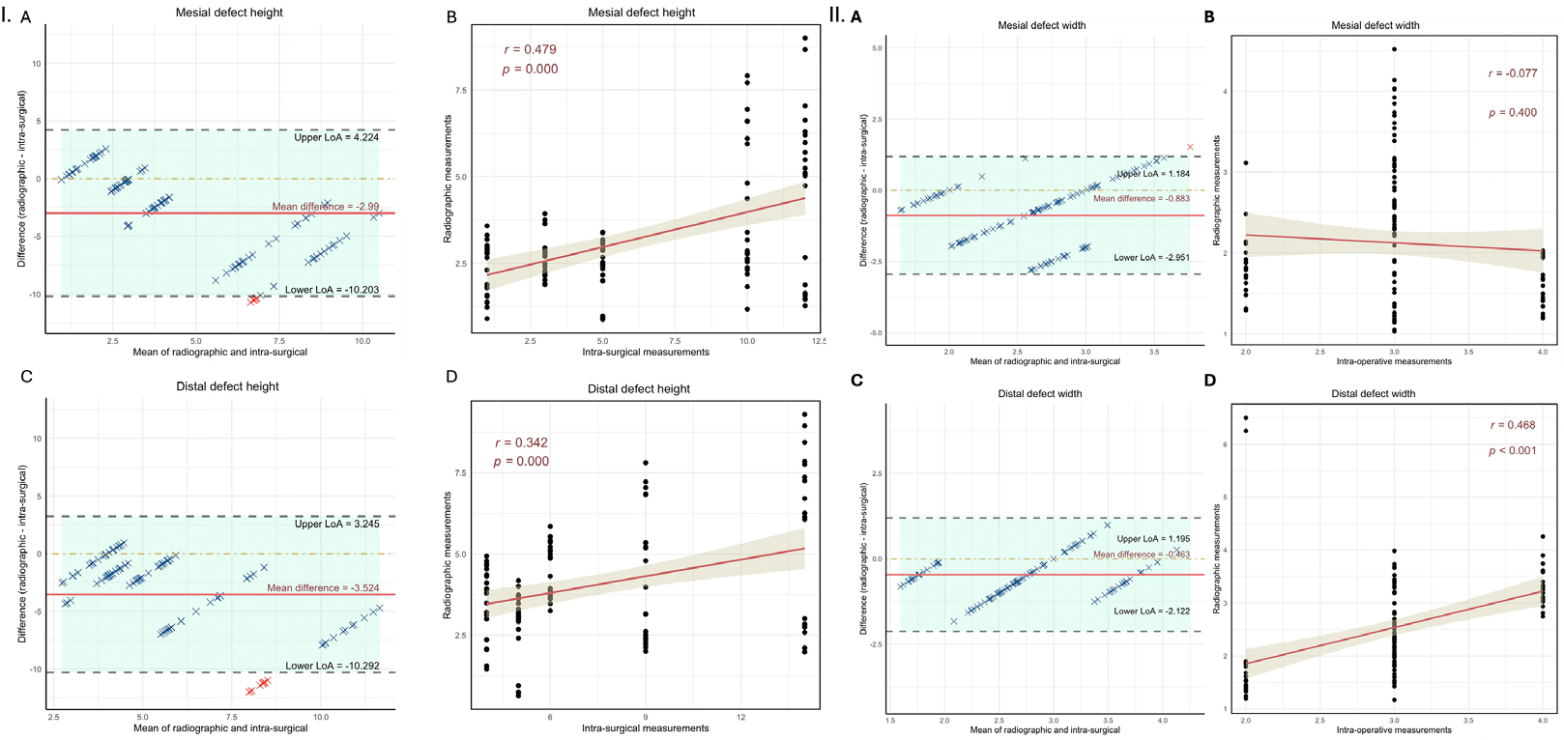
Agreement between calibrated radiographic and intra‐surgical peri‐implant defect measurements. Panels I and II correspond to defect height and defect width, respectively. For each, the Bland–Altman plots (A, mesial; C, distal) display the difference between radiographic and intra‐surgical values (radiographic − intra‐surgical) against their mean, showing the mean difference and 95% limits of agreement; the scatterplots (B, mesial; D, distal) show the corresponding correlation between radiographic and intra‐surgical measurements. (I) Defect height: Mean difference −2.99 mm (mesial; upper/lower LoA 4.224/−10.203) and −3.524 mm (distal; 3.245/−10.292); *r* = 0.479 (mesial) and *r* = 0.342 (distal), both *p* < 0.001. (II) Defect width: Mean difference −0.883 mm (mesial; LoA 1.184/−2.951) and −0.463 mm (distal; 1.195/−2.122); *r* = −0.077 (mesial; *p* = 0.400, not significant) and *r* = 0.468 (distal; *p* < 0.001). Negative mean differences indicate that radiographs underestimated the surgically measured dimensions.

## 4 Discussion

In this exploratory study, calibrated PA radiographs yielded reproducible measurements for EA and DW parameters with clear, well‐defined landmarks whereas DH, DA, and distal bone levels were considerably examiner‐dependent. When compared against the intra‐surgical reference, radiographic DH and DW systematically underestimated the dimensions found at surgery. No clinically reproducible intra‐surgical reference plane exists for EA and EP once the prosthetic restoration is removed therefore their clinical value is difficult to assess; To our knowledge, this is among the first studies to establish the inter‐ and intra‐examiner reliability of EA and emergence profile on calibrated periapical radiographs for both bone and tissue level implants, extending prior work that focused on marginal and crestal bone levels [1, 2]; These differences could reflect the two‐dimensional nature of PA imaging, superimposition of interproximal structures, and more subjective landmark identification [5]. Interestingly, several inter‐examiner ICCs exceeded the corresponding intra‐examiner values, suggesting that averaging readings from multiple calibrated examiners may improve reliability for the less robust parameters. This is consistent with a recent cross‐sectional study reporting high inter‐ and intra‐examiner reliability for radiographic assessment of EA around bone‐ and tissue‐level implants [6].

These findings should be interpreted cautiously due to the limited sample, an examiner pool of only postdoctoral students, and the inability of PA radiographs to capture 3‐D structures. Statistical agreement, moreover, does not necessarily correspond to clinical relevance [7]. Therefore, parameter‐specific conclusions, particularly those graded “poor,” should be regarded as preliminary and hypothesis‐generating rather than definitive; confirmation in an adequately powered, prospective sample is warranted before these estimates inform calibration protocols.

For both retrospective radiographic research and examiner‐calibration protocols, our findings carry two practical implications. First, EA and DW can be reported with reasonable confidence once images are calibrated. Second, DH, DA, and distal bone levels behave as variable measures: they require standardized definitions, parameter‐specific calibration, and averaging across multiple examiners, should be interpreted with caution, and should not be assumed to reflect true dimensions recorded intra‐surgically.

## 5 Conclusions

Within its exploratory limits, this study shows that the reliability of calibrated peri‐implant radiographic measurements may be parameter‐dependent: EA and DW are reproducible, whereas DH, DA, and distal bone levels are examiner‐sensitive and underestimate surgical dimensions. Standardized definitions, parameter‐specific calibration, and multi‐examiner averaging are therefore advisable whenever these less robust parameters are used in clinical research.

## Conflicts of Interest

The authors declare no conflicts of interest.

## Supporting information


**Table S1:** Schematic overview of the examiner training and calibration protocol. Following an initial training phase (Week 1), comprising assessment of examiners' baseline knowledge, a structured tutorial on ImageJ software, and calibration on five training radiographs, examiner calibration was monitored through three repeated measurement sessions of the same five training radiographs, each completed within a 45‐min window, at Weeks 2, 4, and 6, to assess intra‐examiner reliability throughout the study period.
**Table S2:** Operational definitions of radiographic parameters.

**Data S1:** Supporting Information.

**Figure S1:** Radiographs and intra‐surgical photographs measurement of defect height (DH) and defect width (DW) following full‐thickness flap elevation, using a UNC periodontal probe. (A) Defect height, measured from the bone crest to the implant platform (bone‐level implants). (B) Defect width, measured in the plane perpendicular to the implant long axis, between the most coronal point of the endosseous portion of the implant and the bone crest.

## Data Availability

The data that support the findings of this study are available from the corresponding author upon reasonable request.
