# Supplementary material for: Peri‐Implant Radiographic Measurements Compared With Intra‐Surgical Assessments: Diagnostic Accuracy and Reproducibility
Source: J Periodontal Res. 2026 Sep 29;61(9):1089–91. doi: 10.1111/jre.70169 (PMC13768983; doi:10.1111/jre.70169)
Supplement: Supplementary file 1 [file JRE-61-1089-s001.docx]

| Experimental Flow Chart | | | |
| --- | --- | --- | --- |
| Training Phase (Week 1)  **II.B. Treatment Planning and Prognosis**  2 Weeks  **II.B. Treatment Planning and Prognosis**  2 Weeks  **II.B. Treatment Planning and Prognosis**  2 Weeks | **Calibration Phase**  **(2 weeks )** | **Repeat Calibration Session (4 weeks)** | **Repeat Calibration Session (6 weeks)** |
| Assess existing knowledge  Tutorial-Image J software  Calibrate and measure 5 training images | Repeated measurements of 10 PAs over a time period of 45 mins | Repeated measurements of 10 PAs over a time period of 45 mins | Repeated measurements of 10 PAs over a time period of 45 mins  Intra examiner reliability assessment |

**Supplementary Tables**

**Table S1:** Schematic overview of the examiner training and calibration protocol. Following an initial training phase (Week 1), comprising assessment of examiners' baseline knowledge, a structured tutorial on ImageJ software, and calibration on five training radiographs, examiner calibration was monitored through three repeated measurement sessions of the same five training radiographs, each completed within a 45-minute window, at Weeks 2, 4, and 6, to assess intra-examiner reliability throughout the study period.

| Parameter | Operational definition |
| --- | --- |
| Marginal bone level (MBL) | Distance from the implant platform (reference point) to the most coronal point of radiographic bone-to-implant contact, measured parallel to the implant long axis, at the mesial and distal aspects. |
| Crestal bone level (CBL) | Distance from the implant platform to the most coronal point of the alveolar bone crest, measured parallel to the implant long axis, at the mesial and distal aspects. |
| Emergence angle (EA) | Angle formed between the implant long axis and the tangent to the restoration contour at the mucosal margin, measured at the mesial and distal aspects. |
| Emergence profile (EP) | Categorical classification of the transmucosal restoration contour (e.g., convex, straight, concave) at the mesial and distal aspects. |
| Defect width (DW) | Horizontal distance, in the plane perpendicular to the implant long axis, between the implant surface and the bone defect margin at the level of the bone crest. |
| Defect height (DH) | Vertical distance, parallel to the implant long axis, from the bone crest to the implant platform (bone-level) or to the transition to the endosseous portion of the fixture (tissue-level). |
| Defect angle (DA) | Angle formed at the apex of the peri-implant bone defect between the implant surface and the sloping bone defect wall. |

**Table S2:** Operational definitions of radiographic parameters.
