# Supplementary material for: Peri‐Implant Radiographic Measurements Compared With Intra‐Surgical Assessments: Diagnostic Accuracy and Reproducibility
Source: J Periodontal Res. 2026 Sep 29;61(9):1089–91. doi: 10.1111/jre.70169 (PMC13768983; doi:10.1111/jre.70169)
Supplement: Supplementary file 2 [file JRE-61-1089-s002.docx]

**Figure S1:** Radiographs and intra-surgical photographs measurement of defect height (DH) and defect width (DW) following full-thickness flap elevation, using a UNC periodontal probe. (A) Defect height, measured from the bone crest to the implant platform (bone-level implants). (B) Defect width, measured in the plane perpendicular to the implant long axis, between the most coronal point of the endosseous portion of the implant and the bone crest.
