# Supplementary material for: Peri‐Implant Radiographic Measurements Compared With Intra‐Surgical Assessments: Diagnostic Accuracy and Reproducibility
Source: J Periodontal Res. 2026 Sep 29;61(9):1089–91. doi: 10.1111/jre.70169 (PMC13768983; doi:10.1111/jre.70169)
Supplement: Supplementary file 3 [file JRE-61-1089-s003.docx]

**Supporting Information**

*Training and calibration protocol.*

Eight graduate periodontics research fellows in the Department of Periodontics and Oral Medicine, University of Michigan School of Dentistry, participated as examiners. Training radiographs (n = 5, separate from the 10 study radiographs) were selected for clear identification of the implant platform as a reference point, with distinct visualization of implant threads, to optimize assessment of bone levels relative to the platform. During training, all seven parameters (Marginal Bone Level, Crestal Bone level, Emergence Angle, Emergence Profile, Defect Width, Defect Height, Defect Angle) were defined and demonstrated by an expert examiner (H-L.W.) using representative radiographs. Following training, examiners independently measured the 10 study radiographs across three sessions at 2-week intervals, blinded to their own and to one another's previous values; each parameter was measured three times per session and averaged. Two investigators (E.K., P.H.) compiled the individual measurement files for statistical analysis. All measurements were performed using ImageJ software (version 1.53t; National Institutes of Health, Bethesda, MD, USA;). Each radiograph was individually calibrated prior to measurement using the ratio of the measured to the known implant length, to correct for projection-related distortion.

*Statistical modeling*

All analyses were conducted by an author with biostatistics expertise (P.H.) using RStudio (version 2025.05.0+496; Posit, PBC) with the R packages *dplyr*, *tidyr*, *irr*, *psych*, and *lpSolve*. Two-way mixed-effects models were used to calculate intraclass correlation coefficients (ICC) for continuous variables. For inter-examiner agreement, measurement session and radiograph ID were modeled as random effects; for intra-examiner consistency, examiner and radiograph ID were modeled as random effects. Fleiss' κ was calculated for categorical variables (emergence profile; bone- vs tissue-level connection). Agreement between radiographic and intra-surgical measurements (DW, DH) was assessed using the Pearson correlation coefficient, mean absolute error, and Bland–Altman analysis. Agreement/reliability values were interpreted according to Cicchetti and Sparrow and Fleiss (a simplified version of the Landis and Koch criteria): <0.40, poor; 0.40–0.59, fair; 0.60–0.74, good; 0.75–1.00, excellent. All tests were two-sided, with significance set at α = 0.05.

*Intra-surgical reference measurements.*

Intra-surgical measurements of Defect Width and Defect Height were obtained at the time of surgical re-entry with a UNC periodontal probe *(Hu-Friedy Mfg. Co, Chicago, IL, USA)* following full-thickness flap elevation and granulation tissue removal. Defect Height was measured from the bone crest to the implant platform in bone-level implants, or to the transition to the endosseous portion of the fixture in tissue-level implants; Defect Width was measured in the plane perpendicular to the implant long axis, between the most coronal point of the endosseous portion of the implant and the bone crest.
